# A Novel Benchmark Dataset for COVID-19 Detection during Third Wave in Pakistan

**DOI:** 10.1155/2022/6354579

**Published:** 2022-08-12

**Authors:** Zunera Jalil, Ahmed Abbasi, Abdul Rehman Javed, Muhammad Badruddin Khan, Mozaherul Hoque Abul Hasanat, Abdullah AlTameem, Mohammed AlKhathami, Abdul Khader Jilani Saudagar

**Affiliations:** ^1^Department of Cyber Security, PAF Complex E-9, Air University, Islamabad, Pakistan; ^2^Information Systems Department, College of Computer and Information Sciences, Imam Mohammad Ibn Saud Islamic University (IMSIU), Riyadh, Saudi Arabia

## Abstract

Coronavirus (COVID-19) is a highly severe infection caused by the severe acute respiratory coronavirus 2 (SARS-CoV-2). The polymerase chain reaction (PCR) test is essential to confirm the COVID-19 infection, but it has certain limitations, including paucity of reagents, is computationally time-consuming, and requires expert clinicians. Clinicians suggest that the PCR test is not a reliable automated COVID-19 patient detection system. This study proposed a machine learning-based approach to evaluate the PCR role in COVID-19 detection. We collect real data containing 603 COVID-19 samples from the Pakistan Institute of Medical Sciences (PIMS) Hospital in Islamabad, Pakistan, during the third COVID-19 wave. The experiments are separated into two sets. The first set comprises 24 features, including PCR test results, whereas the second comprises 24 features without PCR test. The findings demonstrate that the decision tree achieves the best detection rate for positive and negative COVID-19 patients in both scenarios. The findings reveal that PCR does not contribute to detecting COVID-19 patients. The findings also aid in the early detection of COVID-19, mainly when PCR test results are insufficient for diagnosing COVID-19 and help developing countries with a paucity of PCR tests and specialist facilities.

## 1. Introduction

Coronavirus is a highly severe infection caused by the severe acute respiratory coronavirus 2 (SARS-CoV-2) [1]. It has quickly spread across the world [2–4]. It is a highly lethal disease and caused more than 500,000 deaths in only 216 countries. It affects daily human activity, and early detection of this virus is critical to preventing the spread of this contagious virus. COVID-19 may now be detected using the reverse transcription polymerase chain reaction (RT-PCR) test and the rapid antigen testing (RAT) test [5, 6]. These tests are not 100% reliable in identifying COVID-19; 20% of false positives have been recorded, and the method is very time-consuming [7]. The RAT test can identify antibodies to lgM and lgG. It has a sensitivity score of 18.8% and a specificity score of 78.1%, which are the main drawbacks. The majority of underdeveloped nations cannot efficiently execute these tests. Consequently, additional components and testing methods that are more readily available and need fewer computing resources are being promoted [8].

The use of artificial intelligence is growing in popularity all the time [9]. It addresses machine learning (ML), algorithm design, and statistical analysis (among others). It also contributes significantly to clinical and academic research [10, 11]. In the future, ML can be applied efficiently in a variety of domains, including health and medical [12–14], engineering [15–18], sociology [19–21], and others. Machine learning (ML) has been applied in a range of applications, such as COVID-19 detection, tumor detection, and health assessment [22–25].

Pakistan is one of those most affected countries by COVID-19, with 2,53,604 COVID-19-positive cases [26, 27]. The first COVID-19 report was published in Pakistan on February 26, 2020. According to this research, three COVID-19 instances appeared in Pakistan within two days, with no link between these individuals [28]. The number of COVID-19 cases climbed with time, with 1,39,230 positive cases discovered until June 12, after which the aggregate number of cases fell. Until July 25, there were 2,73,113 confirmed case reports.

The COVID-19 outbreak threatens underdeveloped countries like Pakistan's healthcare and medical systems. There are not enough clinicians and medical and healthcare resources in Pakistan to meet these needs. A lack of resources, especially in smaller cities and villages, makes treating patients challenging and reducing mortality rates challenging. Although Pakistan has limited resources and a poor healthcare system, it has effectively enhanced its level of preparedness for COVID-19 [29].

Pakistan is now undergoing three distinct COVID-19 waves. The first wave of COVID-19 arrived in Pakistan in May 2020, with daily increases in COVID-19 confirmed cases and new deaths. It came to an end in the middle of July. The initial wave of COVID-19 had a low mortality rate and went soon, and COVID-19 cases and mortality rates decreased rapidly after peaking.

Pakistan's COVID-19 crisis calmed following the first wave, with fewer new deaths and positive cases. However, at the start of November 2020, the second wave of COVID-19 arrived, and new cases and deaths started increasing. The second is modest in intensity, primarily affecting Sindh's southern province, and reached its peak in the middle of December 2020. The third wave in Pakistan began in the middle of March 2021. When the third wave began, the pattern of mortality rate and COVID-19 cases reached a climax. This wave primarily impacted on Punjab and Khyber Pakhtunkhwa provinces. The third wave reached its apex in April 2021; then, COVID cases and the number of deaths decreased.

- We propose an ML-based approach to detect COVID-19-positive individuals and analyze the effectiveness of the PCR test feature in the COVID-19 detection process.
- We collect a real dataset from the Pakistan Institute of Medical Sciences (PIMS) Hospital, Islamabad, Pakistan, during the third wave, with the assistance of a clinician who understands the advantages and disadvantages of each feature in the dataset.
- Various feature extraction approaches are used to validate the significance of clinician features. For our data, 24 features are identified as the most critical parameters the clinician recommends to detect COVID-19.
- Experiments reveal that the decision tree model outperforms other ML models using and without PCR tests.

Section 2 discusses prior studies on the subject. The dataset is described in Section 3. The suggested approach is presented in Section 4.  Section 5 presents and evaluates the experimental setup and results.  Section 6 contains the conclusion and future work.

## 2. Literature Review

This section evaluated several previous works similar to ours. We reviewed the literature based on the different techniques for diagnosing COVID-19, samples obtained, selected samples for analysis, and the constraints observed throughout the study. PCR test is performed when the RNA is extracted correctly using a specified clinical methodology. This is currently a one-of-a-kind approach for diagnosis. Unfortunately, there are still many constraints on it. This test necessitates using advanced equipment and skilled personnel [30]. Testing a single sample is impractical due to the high cost and time required, almost 4 to 5 hours. PCR machines are utilized with a set of testing samples to preserve expense. The incorrect results are found at a rate expected to range from 3 to 30% [31]. This false-negative rate is risky since the patient is not isolated and may contribute to the spread of this disease. In addition to PCR testing, CT scans can be used to detect this virus [32, 33]. Unfortunately, CT scan is unable to detect a precise diagnosis of this infection. It is also not easily accessible everywhere and might expose patients to needless radiation [34]. As a result, clinicians do not advise CT scans and chest radiographs (CXR) for all patients [35]. Clinical and standard blood tests can be utilized to identify COVID-19 affordable and timely manner. Various researches are available that use single, multiple models in a research or a combination of different models. Several studies have utilized various ML models to diagnose COVID-19.

Authors in [36] used routine blood parameters to train the ML model and diagnose COVID-19. They employed 11 of the original 49 parameters. This study included 235 patients, of which 105 were confirmed, COVID-19 patients. They used accuracy, specificity, and sensitivity assessment measures and obtained 95.95% accuracy, 95.13% specificity, and 96% sensitivity, respectively. The suggested research by the authors focuses on early diagnosis by prompt treatment. A random forest technique is used to mine the researcher's work on 11 major blood indicators. The equipment required for a commercial blood test (CBT) to generate 49 blood test samples is then utilized to construct the tool set for assistant discrimination. The critical problem encountered in this investigation is that the need to identify COVID-19 instances with a common symptom is not validated since these cases were difficult to get in the current environment.

Authors in [37] proposed the ML technique and used LR and RF models to detect COVID-19. The data come from 52 COVID-19-infected patients whose CT scans were taken from 5 hospitals in China from January 23, 2020, to February 8, 2020. The models offered properly compute the length of stay in the hospital for patients with COVID-19 pneumonia [38]. The outcome indicates that the patient has a minimum hospital stay of fewer than 10 days or a maximum hospital stay of more than 10 days. Authors in [39] advised using a chest X-ray (CXR) since it is less costly, quicker, and more commonly used. This study uses X-ray imaging to distinguish COVID-19-induced pneumonia from other types. Seven 1144 X-ray images from seven distinct classes are included in this study. This study, however, does not entail a conclusive COVID-19 diagnosis, but it does aid in screening patients in emergency care.

Authors in [40] collected the dataset from San Rafael Hospital. The blood parameters of 279 patients, of which 177 are confirmed, are COVID-19 patients. The most critical characteristics for diagnosis of COVID-19 were aspartate aminotransferase (AST), lactate dehydrogenase (LDH), lymphocyte count (LC), C-reactive protein (CRP), and white blood cell (WBC) out of a total of 279. Various ML models in this study attained accuracy ranging from 82% to 86%.

Authors in [41] used several machine learning models to predict the presence of coronavirus. They used RF, DNN, and XGB models for classification, and the XGB model achieved the highest results compared with RF and DNN model results. This study involves data from 160 COVID-19 patients from Slovenia's University Medical Centre Ljubljana. Furthermore, 5,333 more COVID-19-negative patient data were added to the dataset.

Compared to previous studies, we concentrate on proposing a strategy for early diagnosis of COVID-19 in Pakistan and analyzing the contribution of PCR test in COVID-19 detection. The proposed approach selects the best attributes for early COVID-19 diagnosis using machine learning methods. We utilized a dataset of 603 inpatients in age, gender, and comorbidity.  Table 1 summarizes the remaining articles and their primary characteristics.

## 3. Dataset and Preliminaries

This section describes the overall data collection procedure for positive and negative COVID-19 patients from the Pakistan Institute of Medical Sciences (PIMS) Hospital. From August 2021 to December 2021, we gathered data on inpatients for five months. We collect the dataset every week. During the data collection phase, we go through various challenges. One of the primary challenges is choosing the crucial features that have a part in determining a COVID-19-positive patient. For this purpose, we took advice from a healthcare specialist. Finally, with the assistance of a clinical specialist, we completed the process of dataset selection. Initially, our team collected the dataset in hardcopy form, and then we selected only those columns referred by the clinical expert and finalized our dataset.

**Figure 1:**
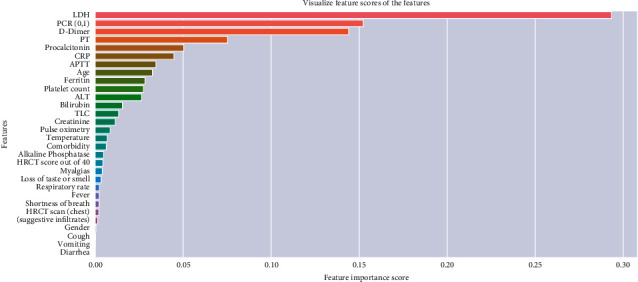
Dataset characteristics and important features for COVID-19.

This dataset contains demographic information, various symptoms, HRCT scan, blood test, and disease histories for 603 inpatients of various genders and ages. The dataset contains 403 COVID-19-positive patients and 200 COVID-19-negative patients. Initially, we had 38 columns in our dataset, and on the suggestion of a clinical expert, we eliminated five columns from the dataset (“Pulse,” “Serum Albumin,” “ *|* BP systolic,” “HB,” and “BP systolic”) since these columns do not help for COVID-19 detection. The clinical expert's feature importance of each column is present in Figure 1.

## 4. Proposed Approach

This research presents a unique ML classifier training and feature selection technique for identifying relevant factors in COVID-19 patients. Collecting COVID-19 patient datasets, choosing features indicated by clinical experts, data analysis, co-relation analysis, feature extraction, model selection, and final prediction is all part of the proposed technique. This section discusses the methodology recommended for this research investigation.

**Figure 2:**
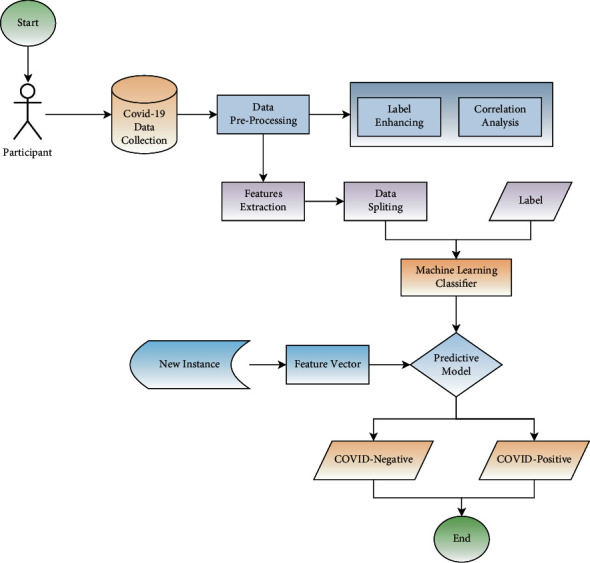
Proposed approach for prediction of COVID-19 in Pakistan.

Figure 2 depicts an overview of the suggested method. Initially, we collected the dataset with the help of a clinical expert. The next step is to turn the hardcopy data into a soft copy or CSV format. The three main phases in data preparation are eliminating irrelevant characteristics, removing missing values, and label encoding. COVID-19 severity and nonseverity may be determined by a patient's first symptoms and clinical expert-selected characteristics. Selecting the most critical characteristics to feed into the machine learning model is accomplished using feature selection algorithms to limit the dataset.

Five prominent ML classifiers, including multi-layer perceptron (MLP), K-nearest neighbor (KNN), support vector machines (SVMs), Naive Bayes (NB), and decision tree (DT), combined with a unique feature selection procedure were utilized to detect positive and negative COVID-19 patients. Model prediction on new unseen data is made after several ML models have been trained on training data. There are numerous approaches in the literature for feature reduction, like using all feasible subsets, forward selection, or backward elimination, and some transformation-based techniques like PCA, fuzzy c-mean, ICA, and their different versions. Unfortunately, these approaches have flaws that require a proper statistical analysis [49–51]. For example, to select the optimal feature subset, all possible subset technique requires 2p rounds, where *p* refers to the number of features. In this study, we engage a clinical expert who understands the benefits and drawbacks of each feature in a dataset to select optimum features. We also incorporate other relevant feature extraction approaches, such as random forest features, extreme gradient boosting (XGB) features, CatBoost features, and chi-square features. We compare features derived by different feature extraction techniques to features specified by a clinical expert, and the random forest feature extraction approach retrieves the most suitable features, which aid the ML classifier in COVID-19 identification. The comparison of all the feature extraction techniques is presented in Table 2.

### 4.1. Preprocessing and Co-Relation Analysis

Data preprocessing is essential in machine learning because the quality of the data and the meaningful information taken from it directly influence our model's ability to learn. As a result, data preprocessing is an important stage in machine learning. The three critical processes in data preprocessing are imputation of missing values, removing nonuseful features, and label encoding using the category code technique. We discovered that our dataset had no null values when doing preprocessing processes. We removed specific nonuseful columns from the dataset that did not contribute to COVID-19 detection in the next preprocessing step. We employed the category code strategy for encoding in the third preprocessing phase. This method transforms category data into numerical numbers. First, we look at the data types of each column because this method needs the category column to be of the “category” data type. So, before employing this method, we alter the data type to “category.”

**Figure 3:**
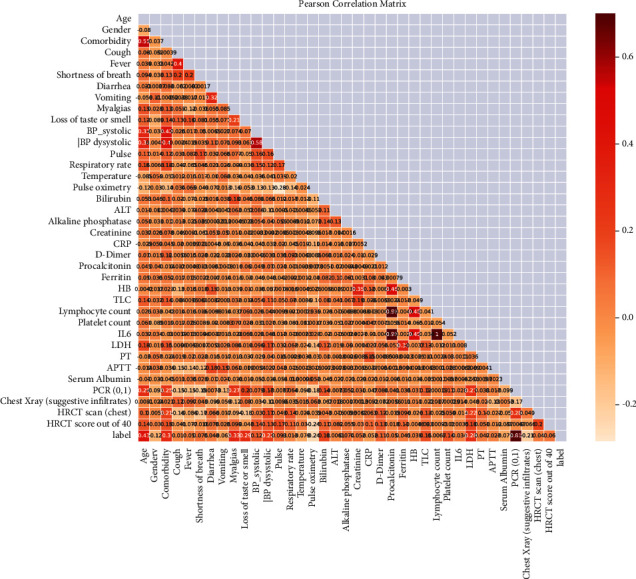
Pearson co-relation feature matrix.

After completing the preprocessing data phase, 33 columns and 603 rows remain. Now we move on to co-relation coefficient analysis. Pearson's coefficient (PCC) is used to examine the co-relationship between the features to eliminate nonessential, duplicate, and redundant features from the data, as shown in Figure 3. The correlation coefficient ranges between −1 and 1. If the value is near −1, the features are adversely associated; if the value is close to 1, the features are closely related and significantly impact model performance. To find the PCC, we set the threshold values to 0.95%; if the correlation value exceeds the threshold, we ignore the feature; if the correlation value is below the threshold, we keep the feature. After the feature co-relation analysis, 31 attributes remain. Consequently, there are few associated characteristics in the dataset; hence, we ignored these features. Therefore, since they are significantly associated or connected, we excluded these columns (“lymphocyte count,” “IL6”) from the dataset.

### 4.2. Feature Selection

The clinical expert understands the benefits and drawbacks of each dataset attribute. The clinical expert evaluates each feature of the dataset and identifies the 24 most important attributes, which are given in Table 2. To identify essential features using artificial intelligence and machine learning-based techniques, we first explored the impact of each feature on severity via feature importance analysis using random forest, XGB, CatBoost, and chi-square feature selection techniques. We then compared the results of these techniques to the clinical expert-selected features. After analyzing the feature importance scores from each feature selection technique, we compared the top 24 features selected by each feature selection approach with clinical expert-selected features. We found that the random forest feature selection technique identifies the essential features that are very similar to the features selected by the expert, as shown in Table 2. Finally, 24 features chosen by a clinical expert and a random forest classifier are fed into machine learning models for COVID-19 detection.

### 4.3. Machine Learning Models

Multi-layer perceptron (MLP), K-nearest neighbor (KNN), support vector machine (SVM), Naive Bayes (NB), and decision tree (DT) models are utilized as cutting-edge prediction models in this study since they are very efficient with unbalanced data.

#### 4.3.1. Multi-Layer Perceptron

MLP is a component of a feed-forward artificial neural network. MLP is well suited to classification and prediction tasks. The MLP model uses the back-propagation technique to reduce the error rate and is a supervised learning method. The simple structure of the MLP model consists of initially three layers: input, hidden, and output layers. MLP consists of a single input layer, single or multiple hidden layers, and finally, one output layer at the end that performs all computations. MLP is a part of supervised learning technique, and it uses function *f*(*Z*) : *R*^*i*^⟶*R*^*n*^ to train on the given data. The total output dimensions are presented by *n*, and the total input dimensions are presented by *i*. We have features set *F*=*f*_1_, *f*_2_ … *f*_24_ with target label *T*_*l*_. Every node is a neuron that performs classification or regression by using a nonlinear activation function. We use most of the default parameters for the MLP model, but some parameter settings are ReLU as activation function, and the learning rate is adam (0.0001) with 200 iterations.

#### 4.3.2. K-Nearest Neighbor

The KNN model is a supervised learning approach that may be used to solve classification and regression tasks. It categorizes data based on numerical outputs. This approach selects the K number of data points from the training data most comparable to the new data point for classification purposes. These neighbors are then utilized to continue the procedure to categorize additional data points. This process is continued until the gap between them is minimal. We performed experiments with 3, 5, and 7 neighbors and observed that the results were almost the same. Thus, we kept the default setting for *n* neighbors. All other parameter values are left at their defaults. We utilized 5 clusters and Euclidean distance to calculate the distance value in this research.

#### 4.3.3. Support Vector Machine

The support vector machine is another fundamental approach each machine learning expert should use in their research. The SVM model is a popular classification algorithm because it produces high accuracy while consuming less computational resources. SVM is a part of supervised learning algorithms used for classification and regression; however, it is frequently used in classification tasks. It is well known due to its ability to identify abnormalities in higher-dimensional data such as audio data. The scikit-learn package is used to construct this approach. The RBF kernel parameter of the SVM model is set to 4 with a gamma value of 0.001, and the probability state is true in the SVM model parameters.

#### 4.3.4. Naive Bayes

The NB model is an ML classifier that makes strong independence assumptions using Bayes theory. It is a combination of multiple probability models with strict independence assumptions. In simple terms, a Naive Bayes classifier asserts that the presence of a single character in a class is unrelated to the occurrence of any other feature. It is employed in various fields, including spam or ham categorization, sentiment classification, COVID-19, and other medical diagnostics. It is frequently used for classification. Aside from its simplicity, Naive Bayes has outperformed even the most sophisticated classification algorithms. In this study, we use the default parameters of the Naive Bayes classifier.

#### 4.3.5. Decision Tree

A decision tree (DT) is a rule-based classifier that recursively splits the dataset until it is left with leaf nodes. DT works based on information gain and entropy measures. Entropy is the impurity that shows how impure a particular node is, and information gain reveals how informative a particular node is to predict the classes. For this study, we set several parameters according to the current scenario. We set cross-validations to 10, and the rest of the parameters are set to default. We set the confidence factor to 1.0.

## 5. Experimental Result and Discussion

This study examined data from 603 patients; 400 were COVID-19-positive, and 203 were COVID-19-negative patients. The dataset is separated into two sections. 80% of the dataset is utilized for training the ML models, while the remaining 20% is used for testing. The selection of a model and its deployment to a dataset to train the model is simply one component. Applying multiple assessment measures to evaluate the model's capabilities on previously unknown datasets is vital in developing any machine learning model. This study included four assessment metrics: accuracy, precision, recall, and F1-score. These assessment measures were determined by using a confusion matrix, with the true positives (TPs) representing COVID-19 patient that is predicted as COVID-19-positive and is COVID-19-positive, the true negatives (TNs) representing COVID-19 patient that is predicted as COVID-19-negative and is COVID-19-negative, the false positives (FPs) representing COVID-19 patient that is predicted as COVID-19-positive but is COVID-19-negative, and the false negatives (FNs) representing COVID-19 patient that is predicted as COVID-19-negative but is COVID-19-positive. The measures employed were as follows.

Accuracy is the ratio of correctly classified COVID-19-positive and COVID-19-negative patients to all correctly and incorrectly classified cases. The equation for accuracy is

(1) $$\begin{array}{c} \text{Acc}=\frac{\text{TP}+\text{TN}}{\text{TP}+\text{TN}+\text{FP}+\text{FN}}. \end{array}$$

Precision is defined as the ratio of a COVID-19 patient successfully detected as COVID-19-positive to all COVID-19-positive occurrences

(2) $$\begin{array}{c} \text{Pre}=\frac{\text{TP}}{\text{FP}+\text{TP}}. \end{array}$$

The recall is defined as the model's sensitivity, which refers to how effectively the classifier can recognize COVID-19-positive patients

(3) $$\begin{array}{c} \text{Rec}=\frac{\text{TP}}{\text{TP}+\text{FN}}. \end{array}$$

The F1-measure is calculated by taking the weighted average of the recall and precision measurements

(4) $$\begin{array}{c} F1-\text{measure}=2\times \frac{\left(\text{Pre}\times \text{Rec}\right)}{\left(\text{Pre}+\text{Rec}\right)}. \end{array}$$

Cross-validation is a typical approach for evaluating ML models' performance and an essential strategy to overcome overfitting. This work utilizes the assessment metrics accuracy, precision, recall, F1-score, and AUC curve score to evaluate model performance. We use a weighted average to evaluate the precision, recall, and F1-score.

This work employed a ten-fold cross-validation approach to assessing our suggested models' performance. The experiments are implemented using Google Colaboratory using Python 3.0. The dataset comprises 38 characteristics; however, we choose just 24 of the best to improve classification results. The classification is performed using ML algorithms, fed with features retrieved during the feature engineering process. The experiments in this study were divided into two phases; in the first phase, we performed experiments that included the PCR test results of patients with other feature sets, and in the second phase, we excluded the PCR test results to determine the importance of the PCR test in diagnosing COVID-19.

**Figure 4:**
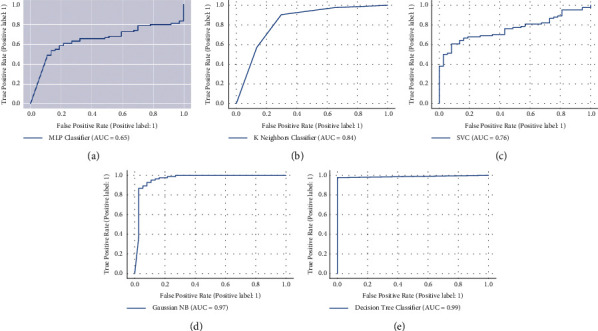
AUC score of machine learning model including PCR test. (a) AUC score of MLP model, (b) AUC score of KNN model, (c) AUC score of SVM model, (d) AUC score of NB model, (e) AUC score of DT model.

The investigation reveals that the experimental findings in both scenarios are almost similar, indicating that the PCR is not the only parameter that indicates whether the patient is COVID-19-positive or not. In many circumstances, such as human mistakes, machine error, and testing samples not obtained appropriately, the PCR result may be inaccurate; thus, it is essential to ensure that the PCR test findings are not the only factor in excluding COVID-19 [52]. Various other characteristics contribute to identifying the existence of COVID-19-positive patients, as indicated in the data selection section. The experimental findings of the suggested technique with the PCR test are shown in Table 3, while those without the PCR test are shown in Table 4. The results in Table 3 demonstrate that the DT model yields the highest accuracy, which is 98.347% compared with other machine learning models (KNN, MLP, SVM, and NB). We also employed precision, recall, and F1-score assessment measures, and the score of these measures using the DT model is 98.134%, 98.245%, and 98.024% in terms of precision, recall, and F1-score. Furthermore, we also compute the AUC score of the DT model, which is 99%. We plot the AUC curve of all models as shown in Figure 4. The AUC curve of the DT model is depicted in Figure 4(e).

**Figure 5:**
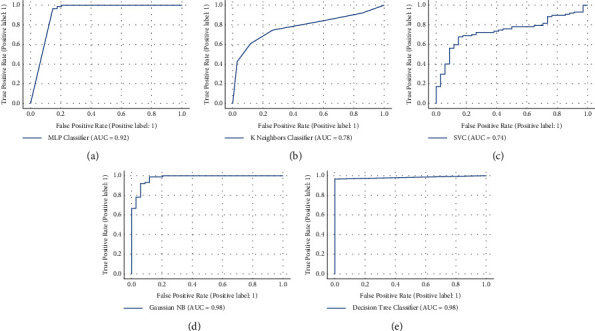
AUC score of machine learning model excluding PCR test. (a) AUC score of MLP model, (b) AUC score of KNN model, (c) AUC score of SVM model, (d) AUC score of NB model, (e) AUC score of DT model.

The findings in Table 4 show that again, in the second phase of experiments, the DT model has the highest accuracy, 96.694%, compared to other machine learning models (KNN, MLP, SVM, and NB). We also use precision, recall, and F1-score evaluation measures in the second phase of experiments. The scores are 97.365%, 97.231%, and 97.153% in precision, recall, and F1-score using the DT model. Furthermore, we compute the AUC score of the DT model, which is 98%. We plot the AUC curve of all models as shown in Figure 5.  Figure 5(e) depicts the AUC curve of the DT model. The experimental findings show that the DT model outperformed other ML models' inaccuracy in both scenarios. The highest AUC curve score demonstrates that the DT model worked admirably on the dataset. As a result, we may deduce that their performance may increase if these algorithms are fed additional data. As we apply the proposed approach to the real dataset to detect this virus, our efforts will benefit the community by analyzing clinical information and taking appropriate steps.

**Figure 6:**
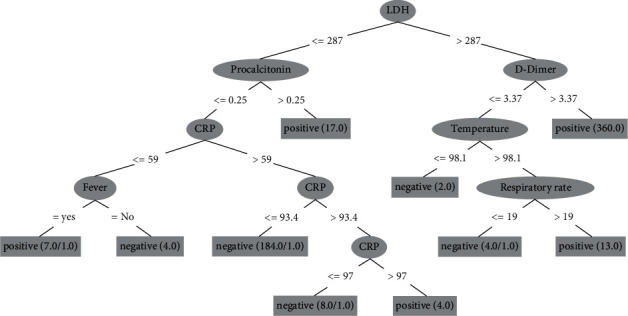
J48 decision tree rules for COVID-19 detection.

The J48 decision tree is depicted in a systematic visual manner in Figure 6. The prediction of positive and negative COVID-19 cases is shown in the decision tree. Ten features participate in predicting COVID-19-positive and COVID-19-negative prediction. LDH is the root node and is considered the best node that provides more information about COVID-19 predicting process. The J48 has 98.70% precision, recall, F1-measure, TP rate, and a 98.90% ROC area. Using a function of the attribute values, the internal nodes with outgoing edges partitioned the instance space into two sub-spaces. The J48 decision tree classifies and forecasts occurrences as positive or negative based on the input factors.

## 6. Conclusion

Most prior research has been limited to chest CT scan and chest X-ray images using DL algorithms to identify positive and negative patients of COVID-19. However, these approaches may accurately diagnose the COVID-19 cases, but these techniques cannot be utilized every time for patients due to high radiations, high prices, and a limited number of available equipment. As a result, distinguishing between positive and negative COVID-19 instances remains a substantial challenge. According to these considerations and based on the previous research analysis, no diagnostic model for identifying COVID-19 cases utilizing numerous clinical features has been developed. As a result, this study aims to use ML classification algorithms to predict COVID-19 patients based on 24 clinical parameters. Five ML models (i.e., MLP, KNN, SVM, NB, and DT) are employed in this study to diagnose COVID-19 patients using 24 clinical features. The models were evaluated using various metrics (accuracy, precision, recall, F1-score, and AUC score). The experiments were carried out in two phases: with PCR test results and without PCR test results. In both cases, the DT classifier outperformed the other four ML classifiers. With an accuracy of 98.347% using PCR and 96.694% without the PCR test attribute, DT is the best classifier for predicting COVID-19 cases based on the 24 clinical variables utilized in this investigation. From a clinical perspective, our findings show that the DT is the best classifier for predicting COVID-19 instances using the 24 variables utilized in this investigation.

When clinicians depend entirely on PCR test results to declare a patient as COVID-19-positive, there is a risk of false-positive and false-negative patients. As a result, disease treatment would be delayed, allowing false-negative patients to spread rapidly. The prediction models might be helpful in early diagnosis, especially when PCR test result is insufficient for diagnosing COVID-19 infection. As a result, this study might help clinicians increase their prediction rate of confirmed COVID-19 infections. The findings are also likely to benefit other countries, especially the developing countries with a scarcity of PCR tests and specialist facilities. The limitation of this research is the less sample size and data balancing issue. In the future, we intend to obtain a more accurate dataset for future studies for better COVID-19 detection.

## Figures and Tables

**Table 1:** List of various approaches to diagnose COVID-19.

Ref.	Dataset source	No. of cases	No. of features	ML model	Highest accuracy (%)	Model AUC score (%)
[42]	Hospital Israelita Albert Einstein	56, 44, 559	24	MLP, SVM, DT, NB	95	-
[43]	Three open-source datasets	279, 1624, 600	15, 34, 19	Extremely randomized trees (ET)	92	85.10
[44]	18 hospitals from Zhejiang, China	914	10	LR, SVM, DT, RF, RL	—	97
[45]	Tongji Hospital, China	413	42	XGB	—	—
[46]	West China Hospital, China	620	9	LR	—	—
[47]	11 regions in China	659	19	DT	89	88
[48]	SMART hospitals	—	—	NB, RF, SVM	93.33	—

**Table 2:** Features extracted from different feature extraction techniques based on importance.

Total features	Random forest	XGB	CatBoost	Chi-square	Clinical expert
Age	√	√	√	√	√
Gender	X	√	√	√	X
Comorbidity	√	√	X	√	√
Cough	X	X	X	√	X
Fever	X	X	X	√	X
Shortness of breath	√	X	X	√	√
Diarrhea	X	X	X	√	X
Vomiting	X	X	√	√	X
Myalgias	√	√	√	√	√
Loss of taste or smell	√	√	√	√	√
Respiratory rate	√	√	√	√	√
Temperature	√	√	√	√	√
Pulse oximetry	√	√	√	√	√
Bilirubin	√	√	√	√	√
ALT	√	√	√	√	√
Alkaline phosphatase	√	√	√	√	√
Creatinine	√	√	√	√	√
CRP	√	√	√	√	√
D-Dimer	√	√	√	X	√
Procalcitonin	√	√	√	√	√
Ferritin	√	√	√	X	√
TLC	√	√	√	X	√
Platelet count	√	√	√	X	√
LDH	√	√	√	X	√
PT	√	√	√	X	√
APTT	√	√	√	√	√
PCR (0, 1)	√	√	√	√	√
Chest X-ray (suggestive infiltrates)	X	√	√	√	X
HRCT scan (chest)	√	√	√	√	√
HRCT score out of 40	√	X	X	√	√
Pulse	X	X	X	X	X
Serum albumin	X	X	X	X	X
*|* BP systolic	X	X	X	X	X
HB	X	X	X	X	X
BP systolic	X	X	X	X	X
Lymphocyte count	X	X	X	X	X
IL6	X	X	X	X	X

**Table 3:** Machine learning model result (%) including PCR test.

Model	Accuracy	Precision	Recall	F1-score
MLP	63.632	75.235	64.354	65.356
KNN	82.645	84.156	83.216	83.216
SVM	69.426	48.264	69.203	57.325
NB	71.902	84.320	72.361	73.246
DT	98.347	98.134	98.245	98.024

**Table 4:** Machine learning model result (%) excluding PCR test.

Model	Accuracy	Precision	Recall	F1-score
MLP	55.373	81.262	55.235	56.156
KNN	74.382	78.620	74.165	75.135
SVM	71.902	52.130	72.320	60.233
NB	73.552	86.265	74.360	75.143
DT	96.694	97.365	97.231	97.153

## Data Availability

The data used to support the findings of this study have not been made available because of the agreement with the PIMS Hospital.
